# Characteristics of m^6^A-related LncRNAs in breast cancer as prognostic biomarkers and immunotherapy

**DOI:** 10.7150/jca.87079

**Published:** 2023-09-11

**Authors:** Xinwei Han, Yu Chen, Jiaogui Xie, Yichao Wang

**Affiliations:** 1 Tai Zhou Central Hospital (Taizhou University Hospital), No.999 Donghai Road, Jiaojiang District, Taizhou, Zhejiang, 318000, China.; 2 Cytotherapy Laboratory, Shenzhen People's Hospital, 1017, Dongmen North Road, Luohu, Shenzhen, 518020, China.

**Keywords:** M^6^A, Breast cancer, LncRNA, Prognostic signature, Immune characteristic

## Abstract

N6-methyladenosine (m^6^A) is a common RNA modification in coding and non-coding RNAs and plays an important role in the occurrence and development of breast cancer (BC). However, the role of m^6^A-related lncRNAs in breast cancer prognosis is unclear. This study aimed to help verify the biological function of m^6^A-related lncRNAs in breast cancer prognosis through bio-informatics techniques. First, we screened 18 m^6^A-related lncRNAs from the TCGA database: AL137847.1, AC137932.2, OTUD6B-AS1, MORF4L2-AS1, AC078846.1, AC012442.1, AL118556.1, AL138955.1, AC009754.1, AC024257.4, AL391095.1, AC024270.3, AC087392.1, LINC02649, AC090948.2, AL158212.1, ITGA6-AS1, AL133243.2 and constructed a risk-prognosis model based on this. Based on the model's median risk score, BC patients were divided into high-risk and low-risk groups. Then, the predictive value of the model was verified by Cox regression, Lasso regression, Kaplan-Meier curve and ROC curve analysis, and biological differences between the two groups were verified by GO enrichment analysis, tumor mutation burden, immune indications and in vitro tests. Importantly, the risk score of this prognostic model is an excellent independent prognostic factor, and m^6^A regulators are differentially expressed in patients with different risks. In addition, based on patients' different sensitivities to drugs, some drug candidates for different risk populations are screened to provide targets for breast cancer treatment. The difference in immune function between high-risk and low-risk patients also affected the sensitivity to immunotherapy. In the validation of clinical samples, we analyzed the expression of relevant lncRNAs in different risk groups and speculated the possible impact on the prognosis of breast cancer patients. The risk assessment tool built based on the full analysis of these m^6^A-related genes and m^6^A-related lncRNA libraries, as well as the m^6^A-related lncRNAs, has a high prognostic prediction ability, which may provide a supplementary screening method for accurately judging the prognosis of BC and a new perspective for personalized treatment of breast cancer patients.

## Introduction

Breast cancer has become the most common cancer worldwide and is the fourth leading cause of cancer-related deaths 1. Patients with different stages and types receive different treatment methods, but surgery, radiotherapy, and chemotherapy are still the most effective treatments for breast cancer 2, 3. The prognosis of advanced breast cancer is poor, the 5-year survival rate is less than 57% 2. In addition, advanced breast cancer has higher heterogeneity and a greater risk of metastasis. Its pathological features and treatment methods vary from person to person, and the treatment of surgery, radiotherapy, chemotherapy and immunotherapy made some progress 4. However, breast cancer patients still face the problem of high incidence, high metastasis and high recurrence rate. More importantly, breast cancer is a highly heterogeneous and complex tumor with different biological characteristics and activities in different patients. Specific biomarkers are of great significance for early screening and prognosis of breast cancer. Therefore, the establishment of a prognostic risk model can guide the screening of breast cancer patients and improve the overall survival rate of patients.

As the most abundant RNA modification in eukaryotes, N6-methyladenosine (m^6^A) also plays an important role in the prognostic regulation of cancer 5, 6. M^6^A is a reversible dynamic epigenetic modification, widely occurring in RNA modification (mRNA and ncRNA) 7. Expression levels of m^6^A regulators methylated transferase complex (“writers”), demethylation transferase complex (“erasers”), and function manager (“readers”) are frequently dysregulated in various types of cancers, which may be associated with cancer progression, drug resistance, and prognosis 5. Methyltransferase-like 3(METTL3), METTL14, METTL16, KIAA1429, Wilms tumor 1-associated protein (WTAP), RNA-binding motifprotein 15(RBM15), and zinc finger CCCH domain-containing protein 13 (ZC3H13), as a methylation modification enzyme of m^6^A, can lose methylation modification under the action of demethylation enzyme (obesity-associated protein (FTO) and alkB homolog 5(ALKBH5)). On the contrary, In YTH domain-containing 1 (YTHDC1), YTHDC2, YTH N6-methyladenosine RNA-binding protein 1 (YTHDF1), YTHDF2, YTHDF3 and HNRNPC recognize methylation information to help process RNA 6.

Long non-coding RNAs (lncRNAs) refer to RNAs with low protein-coding potential (ncRNAs) larger than 200bp 8. LncRNA plays an important regulatory role in cell development by affecting gene expression at epigenetic, transcription and post-transcription levels 9. M^6^A modification of long non-coding RNAs regulates the cutting, transportation, stability and degradation of non-coding RNAs themselves, thus affecting a series of biological processes including tumor cell proliferation, metastasis and balance of tumor microenvironment 8. In addition, abnormal m^6^A modifications contribute to the development of cancer. For example, lncRNA TP53TG1 can act on demethylase ALKBH5 to inhibit the development of gastric cancer 10. Overexpression of OTUD6B-AS1 promotes autophagy through lncRNA OtUD6B-AS1/miR-26a-5p/MTDH signalling pathway and thus affects the prognosis of breast cancer patients 11, 12. These m^6^A regulatory factors profoundly affect the occurrence and development of cancers and may be used as early diagnostic markers, prognostic markers and therapeutic targets.

Studies have found that abnormal expression of lncRNAs can be used as a marker for tumor diagnosis, treatment and prognosis 13. However, the function of lncRNAs in m^6^A modification in BC is not completely clear. In this study, we analyzed the role of m^6^A-related lncRNAs in the prognosis of breast cancer and selected 18 m^6^A-related lncRNAs to construct a prognosis model for breast cancer patients, which was further validated and evaluated on training data sets, validation data sets and complete data sets. It is noteworthy that lncRNA can be used as an independent predictor of survival in BC patients without considering other clinical variables. In addition, based on lncRNA expression, different immune functions and sensitivity to immunotherapy among model groups were identified, which further provided a basis for personalized treatment.

## Materials and methods

### Data Acquisition and Analysis

Patient transcriptome data and clinical data were obtained from the Cancer Genome Atlas (TCGA; https://portal.gdc.cancer.gov searches for breast cancer patients) to retrieve raw transcriptome and clinical data information from breast cancer patients. A total of 1226 patients' sample information is included, including 1113 samples of tumor and 113 normal samples, and a total of 1097 samples were analyzed, as shown in **Supplementary Table 1**. According to the m^6^A-related genes in the TCGA database (gene writer: METTL3, METTL14, METTL16, WTAP, VIRMA, ZC3H13, RBM15, RBM15B; reader: YTHDC1, YTHDC2, YTHDF1, YTHDF2, YTHDF3, HNRNPC, FMR1, LRPPRC, HNRNPA2B1, IGFBP1, IGFBP2, IGFBP3, RBMX; eraser: FTO, ALKBH5) defined related lncRNAs, and a total of 1246 related lncRNAs were obtained. Considered as a m^6^A-related by Pearson absolute correlation coefficient >0.4 and a *p* value <0.001 lncRNA) evaluated the correlation between m^6^A-related genes and lncRNA. A total of 20 breast cancer patients were included in the external validation cohort, including detailed clinical information on breast cancer patients in **Supplementary Table 2**.

### Construction of risk model

The clinical information of 1097 patients was obtained in the database. Patients were randomly grouped, and the risk model formula was obtained by train group, and then verified by test group. High and low-risk groups were divided according to the median risk score: lncRNA expression * coefficient (Coef)/sample size, Coef is the coefficient of multivariate Cox regression analysis of 23 lncRNAs, and Exp is the corresponding expression value. Univariate Cox analysis and the Lasso regression model were used to determine lncRNAs associated with patient outcomes in the train group data set. Receiver Operating characteristic Curve (ROC) was used to evaluate the accuracy of lncRNA prognostic characteristics associated with the train data set, test data set, and complete data set.

### Functional evaluation of risk models

Based on the characteristics of relevant lncRNA, the high-risk and low-risk group model constructed by training data set, test data set and complete data set was used for subsequent analysis with clinical information. According to survival analysis, Cox univariate and multivariate analysis (*p* value <0.05), the accuracy of the constructed model and the role of independent prognostic factors in prognosis were determined. Receiver Operating characteristic Curve (ROC) and C-index curve were used to evaluate the ability of the model to predict the prognosis of patients compared with other factors.

### Construction and Validation of Nomogram

Based on lncRNA characteristic risk scores and independent clinical factors, a nomogram prediction model was constructed using R packages. Based on different clinical features, we constructed a risk prediction model to predict 1-year, 3-year and 5-year survival of breast cancer patients.

### Model validation of clinical subgroups

The applicability of the model was verified by grouping different clinical traits. Clinical patients were divided into two groups according to age, one group was less than 65 years old, and the other group was more than or equal to 65 years old. To evaluate the applicability of the model between high and low age groups. Then they were divided into two groups according to different stages: stage Ⅰ and stage Ⅱ was the early stage, stage Ⅲ, and stage Ⅳ was the late stage. The survival difference analysis of risk models of different stages was carried out respectively. If *p* value <0.05 between different groups of the same trait, the survival difference between the high-risk group and low-risk group was considered, which proved that the model constructed by us did not have grouping bias and had high applicability.

### Different genes in the model

In the risk of lncRNA characteristics to build the model, the screen has a significant difference between the high-risk group and low-risk group of genes (| logFC | > 1 and FDR < 0.05), according to the GO enrichment analysis to determine genetic changes have important biological significance. The mutation data of the high and low-risk groups and the mutation burden of tumors were analyzed according to the "MAfTools" R software package and the "GGPUBR" R software package. The survival of patients in the high-risk group and low-risk group combined with the high or low mutation group was analyzed. When *p* value <0.05, the survival of patients in different groups was considered to be different.

### Differences in immune traits among different risk groups

Based on the characteristics of lncRNA risk score and independent clinical factors, construct the risk model using "limma, GSVA, GSEABase, pheatmap, and reshape2" R packages to the risk of immune function difference analysis of the model. The TIDE algorithm was used to analyze the sensitivity of different risk groups to immunotherapy and the possibility of immune escape. The pRRophetic R software package was used to compare drug sensitivity across risk groups to determine which drugs were available. Provide personalized treatment plans for patients.

### Quantitative Real-Time PCR (qRT-PCR)

Clinical samples were obtained from patients with breast cancer from Tianjin Cancer Hospital (Taizhou, Zhejiang Province, China), tumor tissue and corresponding paracancer tissue of 20 patients with breast cancer. Total RNA from breast cancer tissues was extracted by trizol (Thermo Fisher Scientific, China) according to the manufacturer's protocol, and cDNA was synthesized using a reverse transcription kit (Takara, China). qRT-PCR replicates were performed using PowerUpTM SYBR Green Master Mix (Thermo Fisher Scientific, China), and qRT-PCR (ABI-7500, The United States). Primer sequences are available in **Supplementary Table 3**.

### Statistical Analysis

R software (https://www.R-project.org/) and Perl language were used for calculation and statistical analysis. The differences between the high and low risks in the model groups were determined by Kaplan-Meier curves. For descriptive statistics, mean ± standard deviation was used for the continuous variables in a normal distribution. Categorical variables were described by counts and percentages, and *p* value <0.05 in all analyses was considered statistically significant.

## Results

### Identification of m6 A-Associ ate d lncRNAs in Breast Cancer

The regulatory factor of N6-methyladenosine (m^6^A) induces abnormal m^6^A RNA modification, as a common characteristic in the prognosis of breast cancer. To explore the relation between m^6^A RNA methylation regulators and breast cancer, we analyzed 14 m^6^A regulators including writer, reader and eraser in the expression profile of breast cancer during RNA modification. Through the TCGA database, we analyzed 1097 breast cancer samples, lncRNAs associated with m^6^A were screened by Pearson correlation coefficient, selected with absolute correlation coefficient >0.4 and a *p-*value <0.001. All lncRNAs were positively correlated with the co-expression of m^6^A, among the m^6^A regulators, RBM15 has the widest range of effects and can act on about 85% of lncRNAs to regulate breast cancer, METTL3 and YTHDC2 act on about 7% each, and the remaining m^6^A regulators can only regulate about 1% of lncRNAs. (**Supplement Figure S1**).

### Risk model construction of lncRNA charact eristics

**Figure 1:**
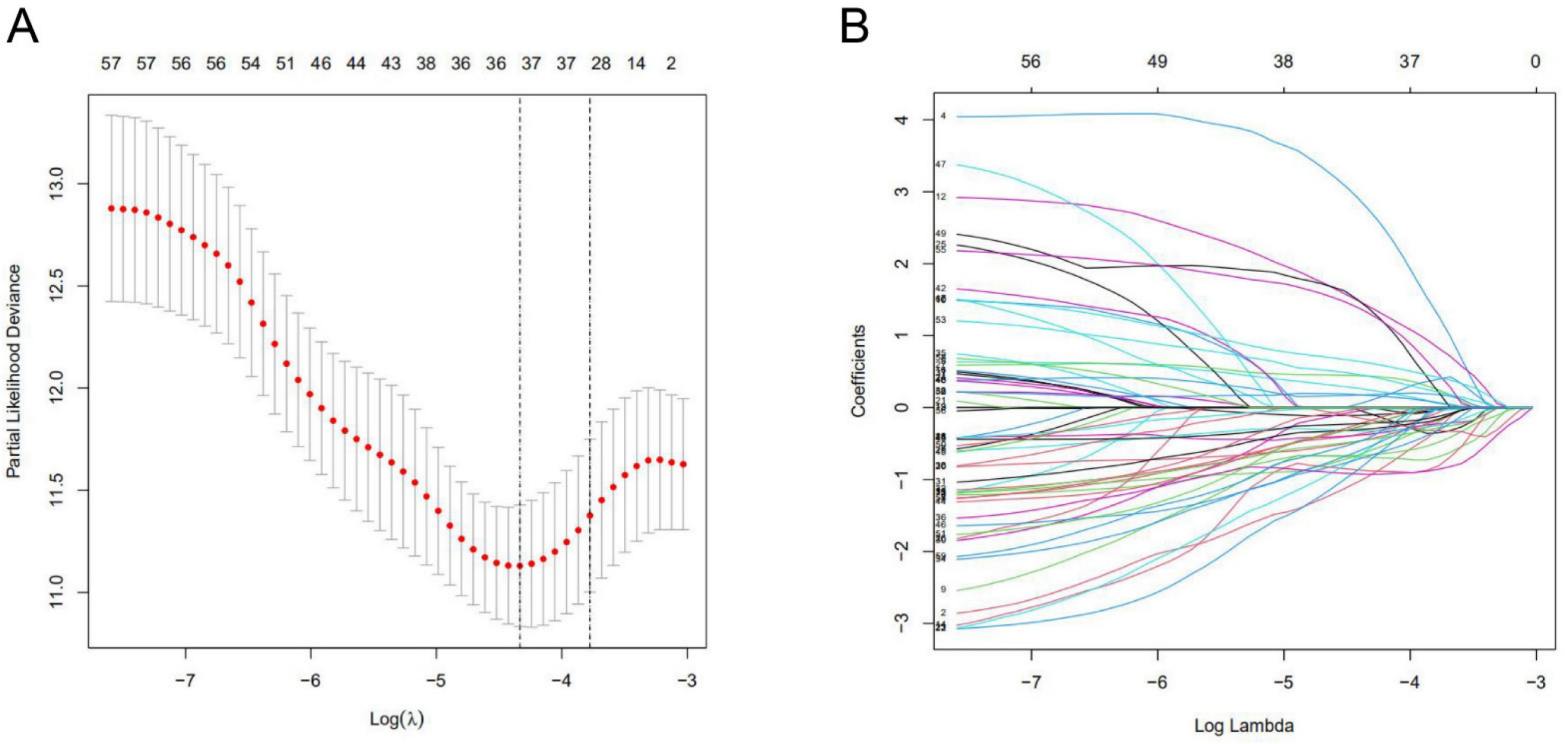
** Characterization of m^6^A-associated lncRNAs signature**. **(A)** 18 lncRNAs used for model construction were analyzed by Lasso regression. **(B)** Selecting the best parameters for BC of the Lasso model (λ).

**Figure 2:**
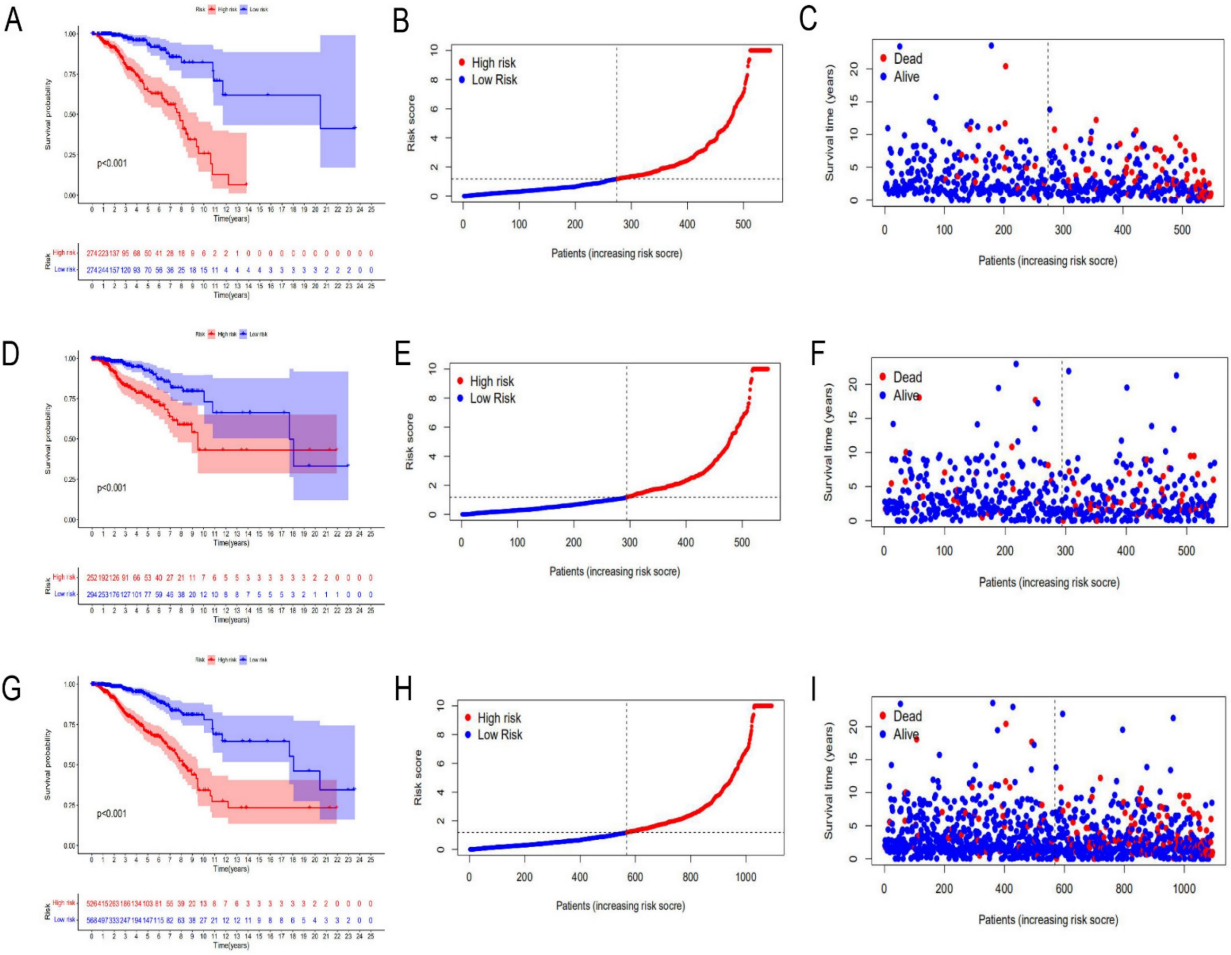
** Risk prognosis model based on m^6^A-related lncRNAs.** Kaplan-Meier curve analysis between the high-risk group and low-risk group was performed in the training data set **(A)**, testing data set **(D)**, and complete data set **(G)**, respectively. Patient risk scores in the high-risk group and low-risk group were shown in the training data set **(B)**, testing data set **(E)**, and complete data set **(H)**, respectively. Patient survivals in the high-risk group and low-risk group were demonstrated in the training data set **(C)**, testing data set **(F)**, and complete data set **(I)**, respectively.

A total of 1097 patient tumor samples were evenly divided into train data sets (N=549) and test data sets (N=548). Then, we performed Lasso regression analysis and univariate Cox regression analysis constructed by 18 m^6^A-related lncRNAs (**Supplement Figure S2** and **Figure 1**) to obtain the median risk in the data set to divide the train data set and test data set and the complete data set was calculated based on the risk formula: risk score = AL137847. 1 * (-2.92970389342707) + AC137932.2 * 5. 13965984463825 + OTUD6B-AS1 * 0.741301698570473 + MORF4L2-AS1 * 3.51751820190694 + AC078846. 1 * (-2.00370072966735) + AC012442. 1 * (-0.953689283417208) + AL118556. 1 * (-2.6686256122187) + AL138955. 1 * (-2.68747823661317) + AC009754. 1 * (- 1.27357342707535) + AC024257.4 * (- 1.66135470206632) + AL391095. 1 * (-0.972178666564357) + AC024270.3 * (- 1.73612202447013) + AC087392. 1 * (- 1. 16409248893925) + LINC02649 * 2.00533615871912 + AC090948.2 * (-0.986934051235532) + AL158212. 1 * 2.22204206572387 + ITGA6-AS1 * (-2. 11197312161232) + AL133243.2 * 1. 14956772567151. Patients in the training data set and testing data set were further grouped into high-risk groups and low-risk groups based on the median risk score. Through the Kaplan-Meier (KM) curve can be seen that the prognosis of the low-risk group based on lncRNA characteristics is better than that of the high-risk group, and the survival time of the low-risk group is significantly higher than that of the high-risk group (**Figure 2**).

### The risk model act as an independent prognostic indicator

The risk model was constructed based on the lncRNA characteristics of patients. Through the correlation heat map, we found that lncRNAs were mainly related to methylase ("writer") METTLE3 and RBM15 in m^6^A and YTHDC2, RBMX and FMR1 that recognize methylation information (" reader ") (**Supplement Figure S3A**), and the main lncRNAs that constitute different risk groups were also different. LncRNA OTUD6B-AS1, MORF4L2-AS1 and LINC02649 were more highly expressed in the high-risk group, while AC024270.3, AC087392. 1 and AC078846. 1 were more highly expressed in the low-risk group. These lncRNAs may be associated with different outcomes in the high-risk and low-risk groups (**Supplement Figure S3B**).

**Figure 3:**
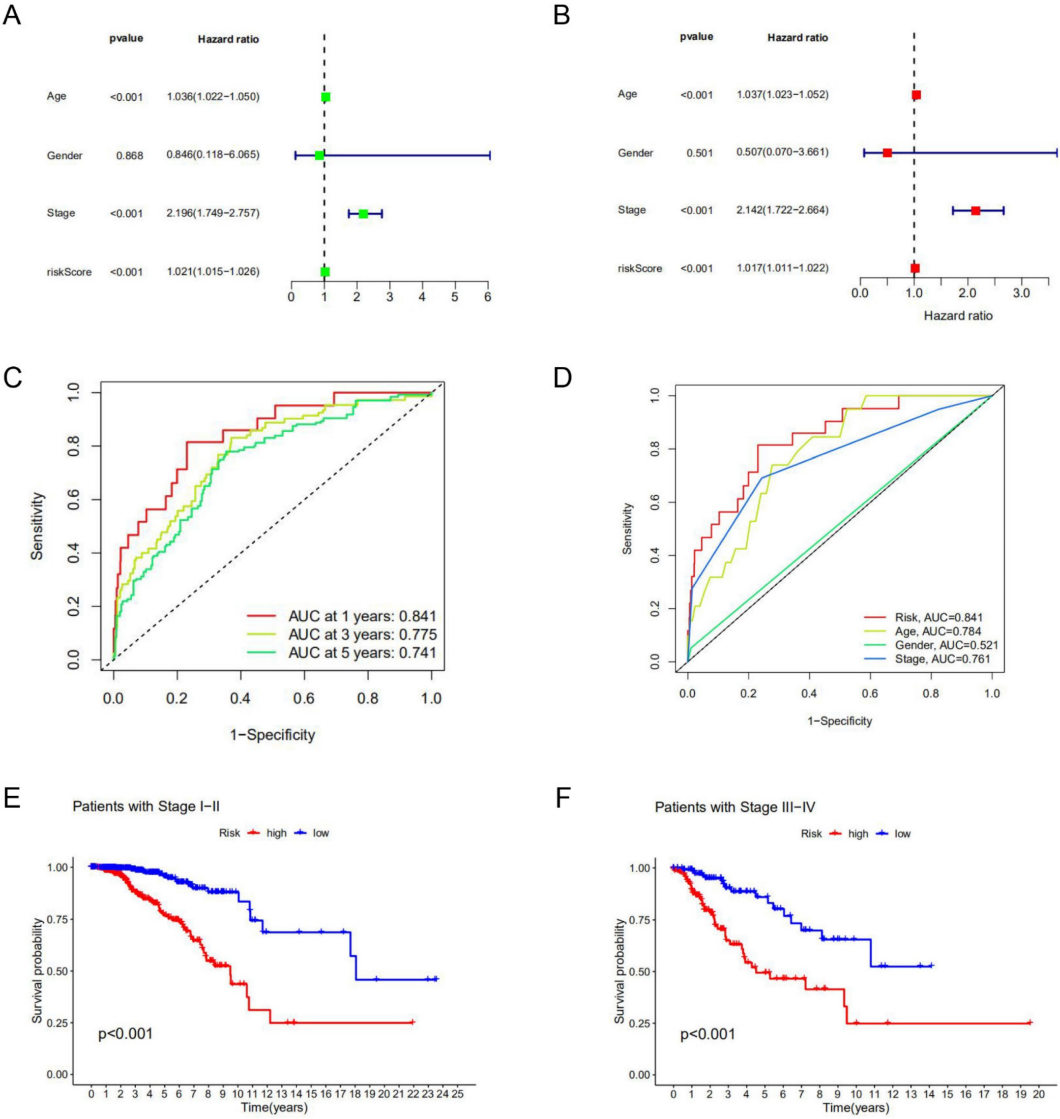
** Prognostic value of the risk model.** Univariate Cox analysis **(A)** and multivariate Cox analysis **(B)** were used to screen independent prognostic factors. **(C, D)** The receiver operating characteristic curve (ROC) was used to assess prognostic factors. **(E, F)** Kaplan-Meier survival curves for high-risk and low-risk groups are stratified by clinical factors of the stage.

**Figure 4:**
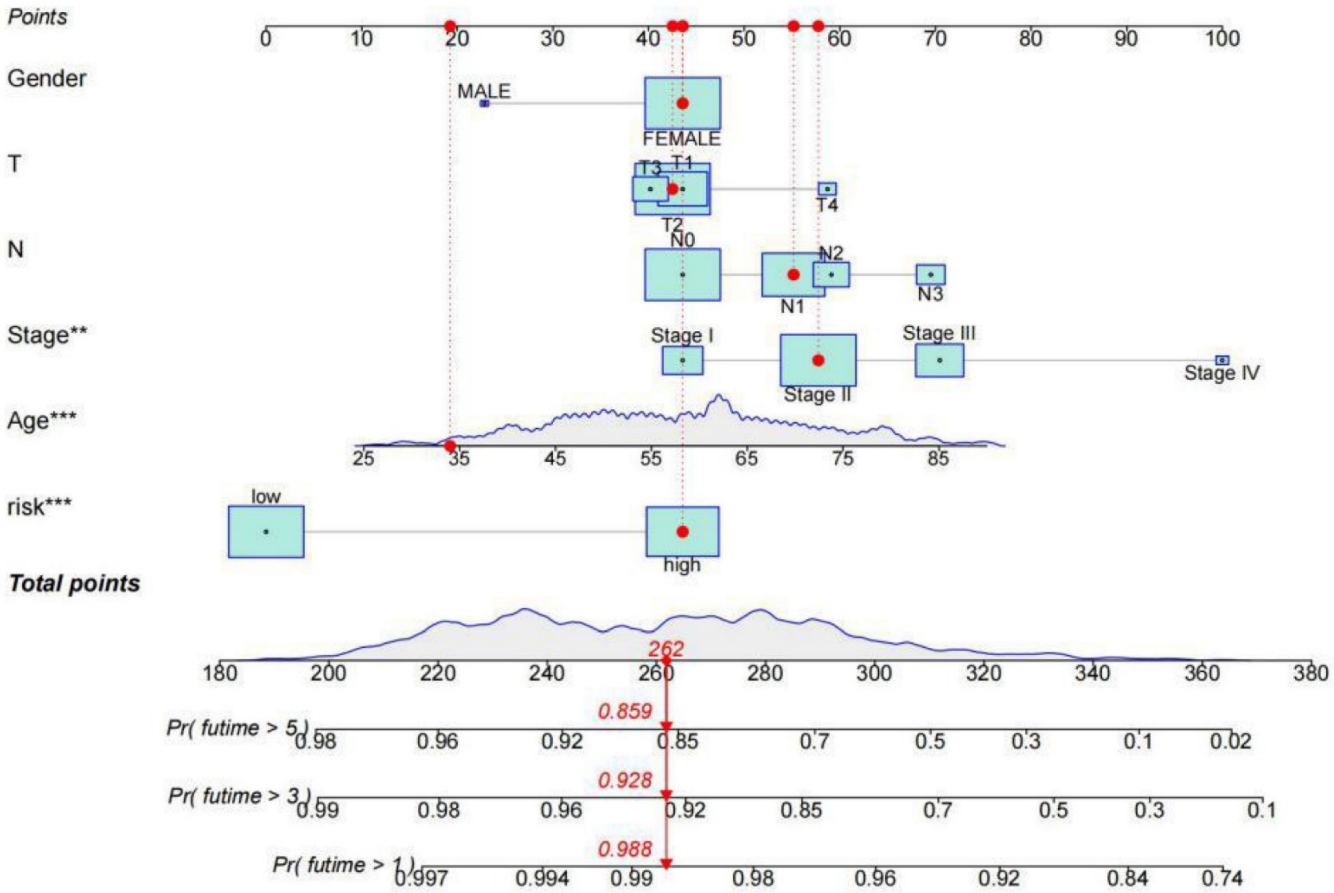
** The survival rate of patients with breast cancer was estimated by rosette.** The scores corresponding to each factor in the line graph are summed to obtain the total score. For example, if the patient had a total score of 262, the 1-year survival rate was 98.8%, the 3-year survival rate was 92.8%, and the 5-year survival rate was 85.9%.

Among clinical factors, age and stage can be used as independent prognostic factors for breast cancer patients. According to univariate Cox regression analysis and multivariate Cox regression analysis, the risk model constructed based on lncRNAs characteristics could also be used as an independent prognostic indicator independent of other factors (*p*<0.05) (**Figure 3A, B**). This factor as a prognostic indicator performs better than other factors (risk score, the area under the curve (AUC) =0.841; age, AUC= 0.784; stage, AUC=0.761; AUCs>0.65) (**Figure 3C**) (**Supplement Figure S4**). The use of risk models can predict 1-year, 3-year, and 5-year survival of breast cancer patients (**Figure 3D**). Kaplan-Meier curve was used to evaluate the association between lncRNA markers and clinical factors, as shown in **Figures 3E and F**. We found that the risk score increased significantly from early stage (stage Ⅰ and Ⅱ) to late stage (stage Ⅲ and Ⅳ) (*p*<0.05). In different clinic-pathological subgroups of breast cancer (age and stage), both early and late-stage patients had lower survival rates in the high-risk group than in the low-risk group. In addition, the younger and older groups showed the same trend (*p*<0.05) (**Supplement Figure S5**). The combination of all effective prognostic factors (including age, stage and risk score) can more accurately predict the survival rate of a patient in one, three and five years (**Figure 4**). These results suggest that the risk score for BC acts well for patient progression prediction, and that m^6^A-associated lncRNA signatures can be used as independent factors to predict prognosis and have a better performance of BC without considering other clinical factors.

### Difference analysis between two risk model groups with lncRNA signature

**Figure 5:**
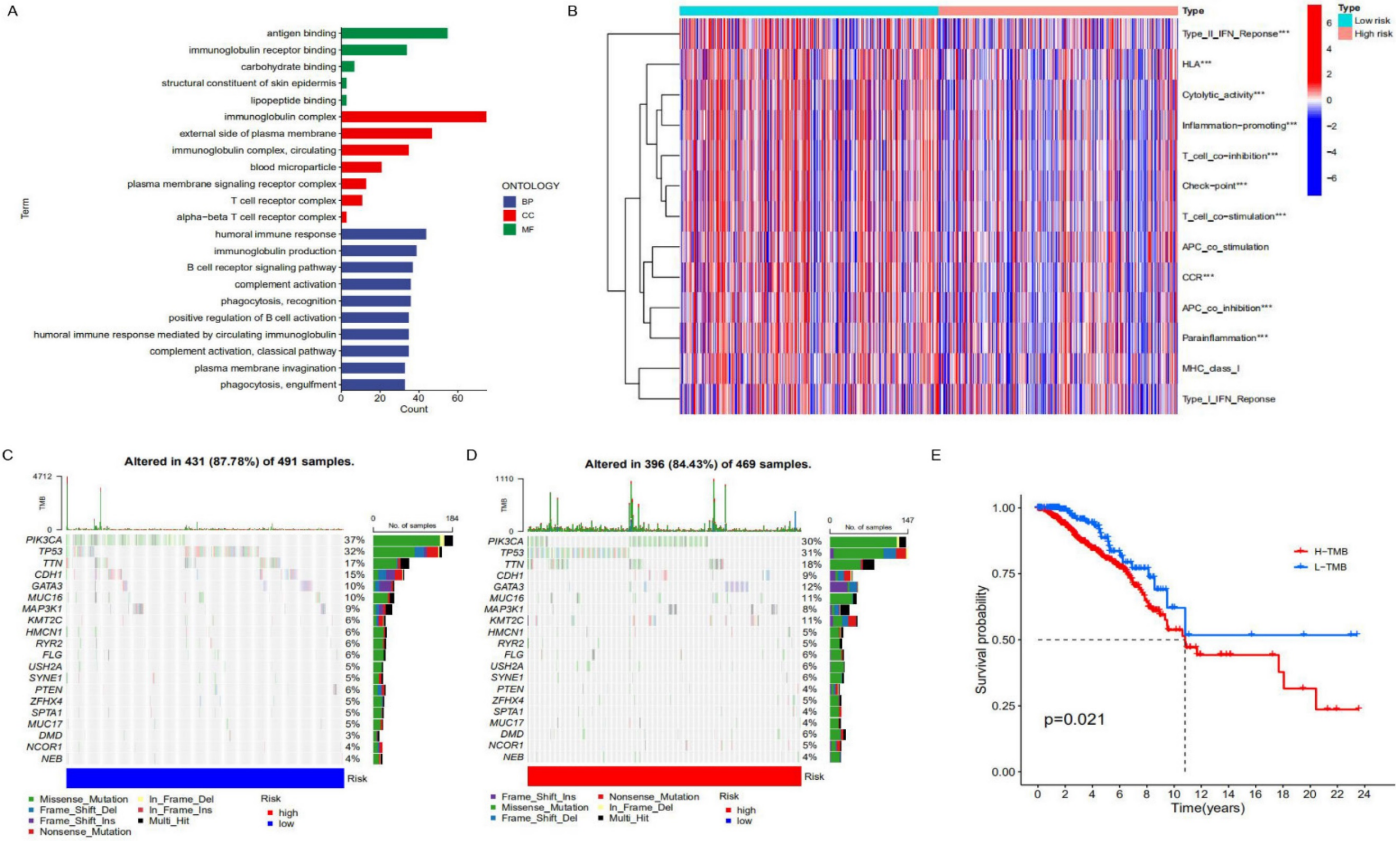
** Analysis of immune characteristics of risk prognostic model. (A)** GO enrichment analysis of differential genes in the high-risk group and low-risk group. **(B)** The relationship of risk groups with different immune traits. **(C, D)** Comparison of tumor mutation burden in the high-risk group and low-risk group. **(E)** Differences in survival rates between the high-risk group and low-risk group characterized by tumor mutation burden (*p*<0.05).

To further clarify the functional differences of specific molecules between low-risk and high-risk groups, differentially expressed genes were identified and functionally annotated via GO. The differentially expressed genes were mainly clustered in multiple important pathways, including Biological Processes (BP): human immune response, immunoglobulin production, B cell receptor signalling pathway, complement activation and plasma membrane invagination etc; Cell Components (CC): immunoglobulin complex, external side of the plasma membrane, immunoglobulin complex, circulating and blood microparticle etc; Molecular Functions (MF): antigen binding, immunoglobulin receptor binding, carbohydrate binding, structural constituent of skin epidermis and lipopeptide binding. As can be seen from **Figure 5A**, the high-risk genes have differences in immunoglobulin complex and circulating in cell components, indicating that there may be differences in immune function between patients in the low-risk group and those in the high-risk group. In addition, there are also differences between the two on the external side of the plasma membrane, so it is speculated that proteins and other components outside the cell membrane may change the migration ability of cancer cells with the change of risk. In terms of molecular functions, differences were observed between antigen binding and immunoglobulin receptor binding, further supporting the hypothesis that there were differences in immune function between the high and low-risk groups (**Figure 5B**). After that, we conducted a Tumor Immune Dysfunction and Exclusion (TIDE) database for the low-risk group constructed with lncRNAs to predict the response of patients to immunotherapy. As can be seen from **Supplement Figure 6**, The TIDE score of the low-risk group was slightly higher than that of the high-risk group, indicating that the low-risk group was more prone to immune escape and less sensitive to immunotherapy than the high-risk group. We speculate that there may be significant differences in immune checkpoint, T cell, and APC cell inhibition between the high-risk and low-risk groups, which makes patients in the low-risk group unsuitable for immunotherapy. Similarly, there are significant differences in humoral immune response, immunoglobulin production, B cell receptor signalling pathway and other immune-related biological processes. In addition, different tumor mutation burdens (TMB) may also be responsible for different immune functions in different risk groups. As a biomarker, tumor mutation burden can help predict patient immunotherapy. We analyzed the difference in tumor mutation burden between the two groups. As shown in **Figures 5C** and **5D**, the tumor mutation burden in the low-risk group was higher than that in the high-risk group, indicating that the low mortality in the low-risk group may be related to the related immune function (**Figure 5E**).

### A personalized treatment pl an based on the established model

**Figure 6:**
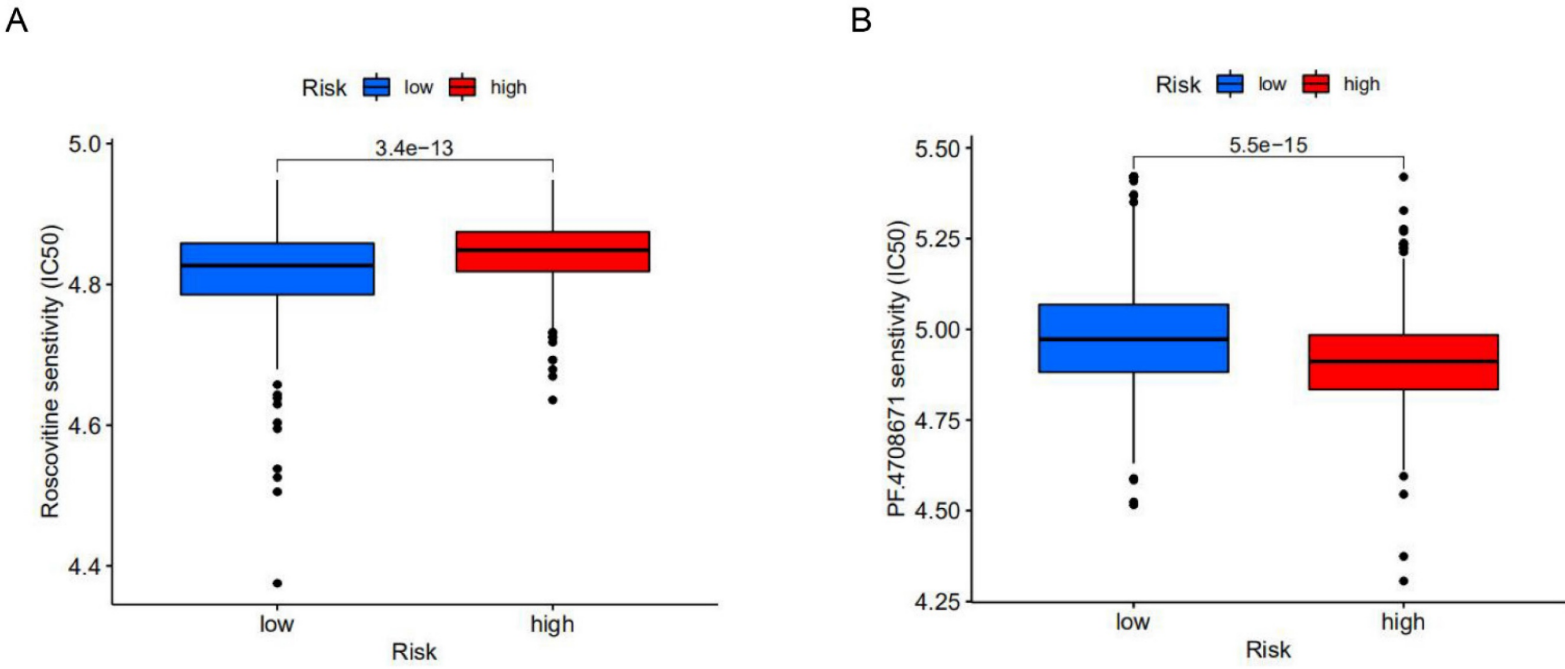
** The effect of different drugs on the risk prognosis model. (A)** The low-risk group was more sensitive to Roscovitine, **(B)** while the high-risk group was more sensitive to PF.4708671.

To explore potential molecular drugs for breast cancer, we used drug-associated pRRophetic R packages based on differential expression analysis between high-risk and low-risk groups. A total of 99 drugs were screened out, among which 84 drugs represented by Roscovitine in the low-risk group were more sensitive than those in the high-risk group, while 15 drugs represented by PF.4708671 in the high-risk group were more sensitive. According to drug trials, Roscovitine is a broad-spectrum purine inhibitor that competes against cyclin-dependent kinases (CDKs) for tumor treatment, while p70S6K, a protein kinase that plays an important role in tumor cells, is inhibited by PF.4708671 for anti-tumour effects (**Figure 6A, B**). This study identified drugs targeting m^6^A-related lncRNA signatures and provided therapeutic targets and ideas for further promoting personalized treatment for patients.

### Validation of the predictive power of risk models in an external clinical cohort

**Figure 7:**
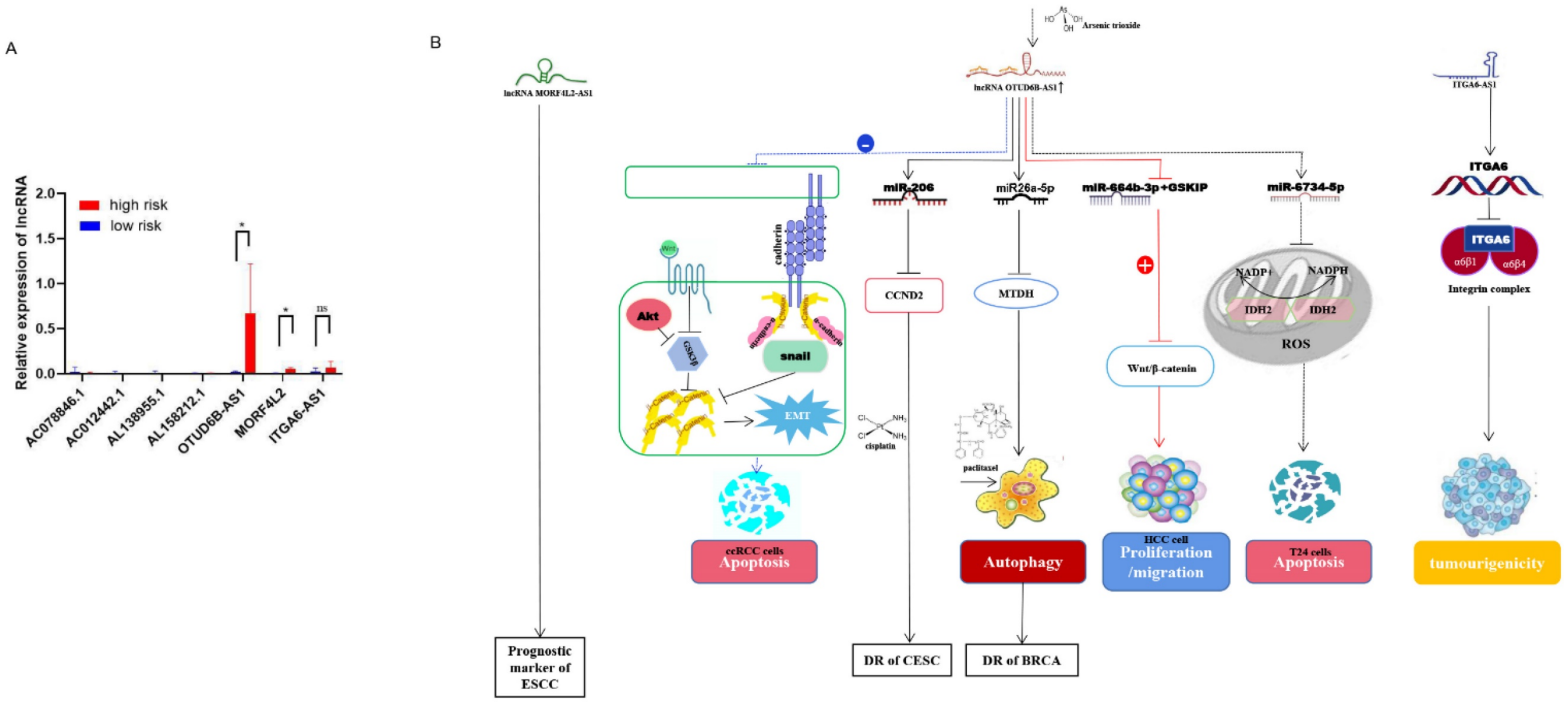
** The expression of m^6^A regulators in different risk models was validated in a clinical cohort. (A)** Expression of m^6^A related lncRNAs in high-risk group and low-risk group, **(B)** lncRNA OTUD6B‐AS1, MORF4L2, ITGA6-AS1 affect cancer development and drug resistance through different signaling pathways.

Validation of the predictive power of the risk model in an external clinical cohort A clinical cohort of 20 BC patients at different stages was established to analyze the role of m^6^A-related lncRNAs in different risk groups. The relative expression of 8 m^6^A-related lncRNAs in 20 breast cancer patients (**Supplementary Table 2**) was analyzed by qRT-PCR (**Supplementary Table 3**). Then, the risk score for each patient was calculated according to the formula (risk = Coef1 * Exp1 + Coef2 * Exp2 + Coef3 * Exp3 + ... + Coefn * Expn, n=8). Among them, Coef was derived from multiple Cox regression coefficients of BC patients in TCGA, while Exp was the expression of m^6^A lncRNAs results of qRT-PCR. A risk score for each patient (n=20) was calculated based on the above formula (**Supplementary Table 4**). Then, we divided 20 BC patients into a high-risk group and a low-risk group (**Figure 7A**). The bioinformatics analysis results were consistent with the qRT-PCR analysis data: AC078846. 1, AC012442. 1 and AC087392. 1 were correlated with the low-risk group, AL138955. 1 and ITGA6-AS1 were not correlated both with the high-risk and low-risk group, and AL158212. 1, OTUD6B-AS1 and MORF4L2-AS1 were correlated with the high-risk group. Long non-coding RNA (lncRNA) OTUD6B anti-sense RNA 1 (OTUD6b-AS1) is a protein-coding gene OTUD6B directed in the anti-sense direction to the relative DNA strand, which is located on chromosome 8 of OTUD6BS1 14, 15. The role of OTUD6B-AS1 in different cancers varies: for example, over-expression of OTUD6B-AS1 in kidney cancer acts on the wnt/β-catenin pathway to inhibit cancer development and metastasis 14; OTUD6B-AS1 is a biomarker of ovarian cancer occurrence and prognosis 16, and high expression of OTUD6B‐AS1 is closely associated with poor prognosis of ovarian cancer 17. Survival rates are lower in hepatocellular carcinoma patients with high OTUD6B‐AS1 expression 18. In breast cancer, over-expression of OTUD6B‐AS1 has been found to promote autophagy and thus genomic instability via the lncRNA OTUD6B-AS1/miR-26a-5p/MTDH signalling pathway, this lncRNA promotes the progression of triple-negative breast cancer 19. Therefore, we speculate that OTUD6B-AS1 plays a similar role in breast cancer and other cancers, and high OTUD6B-AS1 expression will be associated with the poor prognosis of breast cancer patients. Mortality Factor 4 Like 2 (MORF4L2), a component of the NuA4 histone acetyltransferase complex, is involved in the transcriptional activation of selective genes primarily through acetylation of nucleosome histones H4 and H2A 20. It was found that patients with esophagal squamous cell carcinoma with high expression of the MORF4L2-AS1 transcription, as the antisense RNA of MORF4L2, had a poor survival rate 21. The integrin family is an important group of proteins, including integrin alpha (ITGA) and beta proteins. In ITGA, ITGA6, also known as CD49f, is a transmembrane glycoprotein adhesion receptor protein 22. ITGA6-AS1, as an antisense non-coding RNA of ITGA6, can specifically target ITGA6 and lead to its increased expression, thereby regulating the occurrence and aggressiveness of breast cancer and secondary plasmacytic leukaemia 23 (**Figure 7B**). According to the analysis of qRT-PCR, the high expression of MORF4L2-AS1 is correlated with the poor prognosis of breast cancer, but the expression of ITGA6-AS1 does not affect the prognosis of breast cancer, but the specific mechanism of action still needs to be further explored.

## Discussion

Clinical pathological staging is still the gold standard for the diagnosis of breast cancer. However, patients at the same stage also have different prognostic manifestations, which suggests that the pathological staging system has certain limitations in predicting the survival of breast cancer patients. Up to now, emerging research has attempted to construct new and effective strategies to address or complement the limitations of staging systems, including risk models based on tumor-specific lncRNA pattern characteristics, immune infiltration characteristics, and somatic mutation analysis 4, 24. In recent years, lncRNAs have been regarded as new and potential therapeutic targets and biomarkers for cancer treatment, and some studies have proved that m^6^A-related lncRNAs are related to the development of breast cancer 25. However, the role of m^6^A-related lncRNAs in the prognosis and treatment of breast cancer still needs to be further explored.

In the present study, we systematically investigated the role of m^6^A-related lncRNAs in BC prognosis through analysis of tumor sample data from 1113 BC patients in TCGA data set. After identifying associations between 18 m^6^A-related lncRNAs and clinical features, univariate Cox and Lasso regression analyses confirmed that these lncRNAs had independent prognostic value in BC patients. After that, we used the selected 18 m^6^A-related lncRNAs to establish a risk model for predicting the survival of BC patients, which could effectively divide patients into a high-risk group and a low-risk group. Further analysis proved that the risk prediction model of m^6^A-related lncRNAs could be used as an independent risk factor for BC patients with different pathological stages. ROC analysis results showed that the risk model constructed with lncRNA characteristics had high accuracy in predicting the 1-year, 3-year and 5-year survival rates of BC. These results suggest that lncRNA markers are closely related to the prognosis of patients and may be used as biomarkers related to the prognosis of BC. Based on this, we designed a Nomogram combined with a variety of independent prognostic factors to predict patient survival, which may provide patients with a more accurate basis for prognostic survival.

Many studies have shown that m^6^A modification plays a role in the occurrence and development of a variety of cancers, and m^6^A can affect the occurrence and development of tumors and the prognosis of tumor patients by modifying specific lncRNAs. lncRNA THOR, modified by m^6^A, regulates the proliferation of cancer cells in an m^6^A reader-dependent manner 26. In a study of pancreatic ductal adenocarcinoma, lncRNA-PACERR could affect prognosis in patients with pancreatic ductal adenocarcinoma in an m^6^A-dependent manner by binding to IGF2BP2 27. LncRNA CBSLR affects the m^6^A modification of CBS by acting on YTHDF2, and high expression of CBSLR leads to poor prognosis of gastric cancer patients 28. Most of these studies focused on the impact of a single lncRNA on tumor prognosis, but in the process of tumor occurrence, multiple molecules often interact together. In this study, we identified 18 m^6^A-related prognostic lncRNAs in BC and constructed a risk-prognosis model based on these lncRNAs, which will provide more comprehensive predictive information than a single molecule.

In the clinic, it is still difficult to provide personalized treatment according to different patient characteristics. Treatment includes traditional surgery as well as drug therapy and immunotherapy. Tumor mutation burden 29 is an indicator indirectly measuring tumor antigenicity caused by somatic tumor mutation, which can capture the characteristics of T cell activation of patients and serve as an indicator of whether patients are suitable for immunotherapy 30. We conducted gene difference analysis on the constructed model groups, and used GO enrichment analysis to study the differences in biological characteristics between high-risk and low-risk groups. It was found that immunoglobulin complex, outer wall of plasma membrane, antigen binding, immunoglobulin receptor binding, human immune response and immunoglobulin production were more frequent in high-risk groups. We then assessed the differences in immune profiles between the high-risk and low-risk groups. The results showed that T cells and APC cells in the high-risk group had less inhibition of co-activation signals and fewer immune checkpoints. In the tumor mutation burden statistics, we found that the high and low risk groups had similar tumor mutation burdens, suggesting that both the high-risk and low-risk groups may be suitable for immunotherapy. TIDE database analysis indicated similar conclusions, and the high-risk group with a lower TIDE score may have a better therapeutic effect of immune checkpoint inhibitors 31-33. In addition, in the drug database, we screened 99 drugs with different sensitivity to high-risk and low-risk groups (*p*<0.05), mainly inhibitors targeting proteasome, protein kinase, DNA damage-related repair and checkpoint. Of these, 84 were for the low-risk group and 15 were for the high-risk group. It is suggested that when analyzing breast cancer patients in different risk groups, immunotherapy combined with Roscovitine to treat low-risk patients, and immunotherapy combined with PF.4708671 to treat high-risk patients may provide better treatment to extend the survival of breast cancer patients.

Clinical cohort studies have shown that the expression of m^6^A-related lncRNAs used to construct risk prognosis models is different in patients in different groups, which may affect the prognosis of breast cancer patients. Previous studies have shown that high expression of OTUD6b-AS1 is associated with poor prognosis in ovarian cancer patients and high expression of MORF4L2-AS1 in esophageal squamous cell carcinoma patients with poor prognosis, suggesting that these two lncRNAs may contribute to poor prognosis in breast cancer patients through a similar mechanism of action.

In summary, we established a prognostic model in which the expression of m^6^A-related lncRNAs in breast cancer patients has high prognostic value, which can be used as an independent factor to predict the survival rate of patients. Moreover, the feasibility of related immunotherapy and drug therapy was analyzed based on the characteristics of patients in different risk groups. It provides new insights into the role of m^6^A-related lncRNAs in breast cancer and provides ideas for personalized treatment. However, some issues still need to be mentioned for potential clinical translational applications. First, because the data analysed in this study came from open-access online databases and sample validation, the predictive effect of the model in practice and the provision of targeted treatment protocols will need to be validated in the future in collaboration with traditional predictive methods, taking into account multiple influencing factors. Second, since different bio-informatics algorithms may lead to different results, expanding the clinical data set helps to improve the accuracy of the model.

## Supplementary Material

Supplementary tables.

## Abbreviations

AUC: area under the curve
BC: breast cancer
BP: biological processes
BRCA: breast cancer
CC: cell components
ccRCC: clear cell renal cell carcinoma
CESC: cervical squamous cell carcinoma
CDK: cyclin-dependent kinases
DR: drug resistance
ESCC: esophageal squamous cell carcinoma
GO: Gene Ontology
HCC: hepatocellular carcinoma
KM: Kaplan-Meier
MF: molecular functions
MORF4L2: mortality factor 4 like 2
m^6^A: N6-methyladenosine
ROC: receiver operating characteristic curve
TIDE: tumor immune dysfunction and exclusion
TMB: tumor mutation burden
