# Supplementary material for: Characteristics of m6A-related LncRNAs in breast cancer as prognostic biomarkers and immunotherapy
Source: J Cancer. 2023 Sep 11;14(15):2919–30. doi: 10.7150/jca.87079 (PMC10539557; doi:10.7150/jca.87079)
Supplement: Supplementary file 1 — Supplementary tables. [file jcav14p2919s1.pdf]

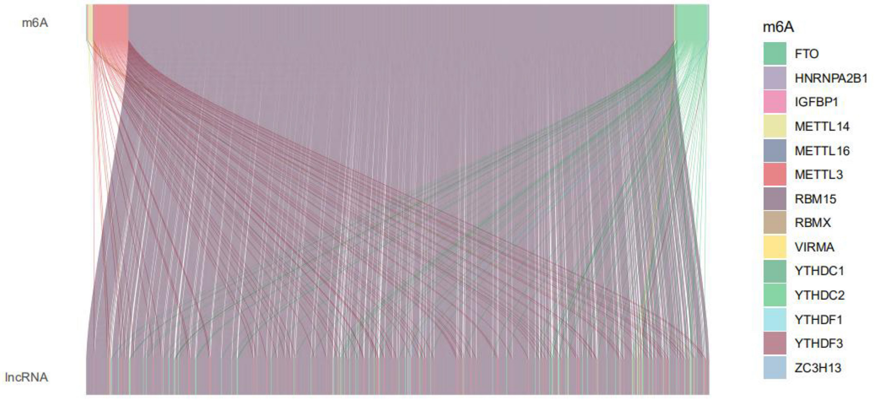

Supplementary Figure 1. Correlation analysis of 14 m^{6}A RNA methylation regulators and lncRNA based on Pearson coefficient analysis.

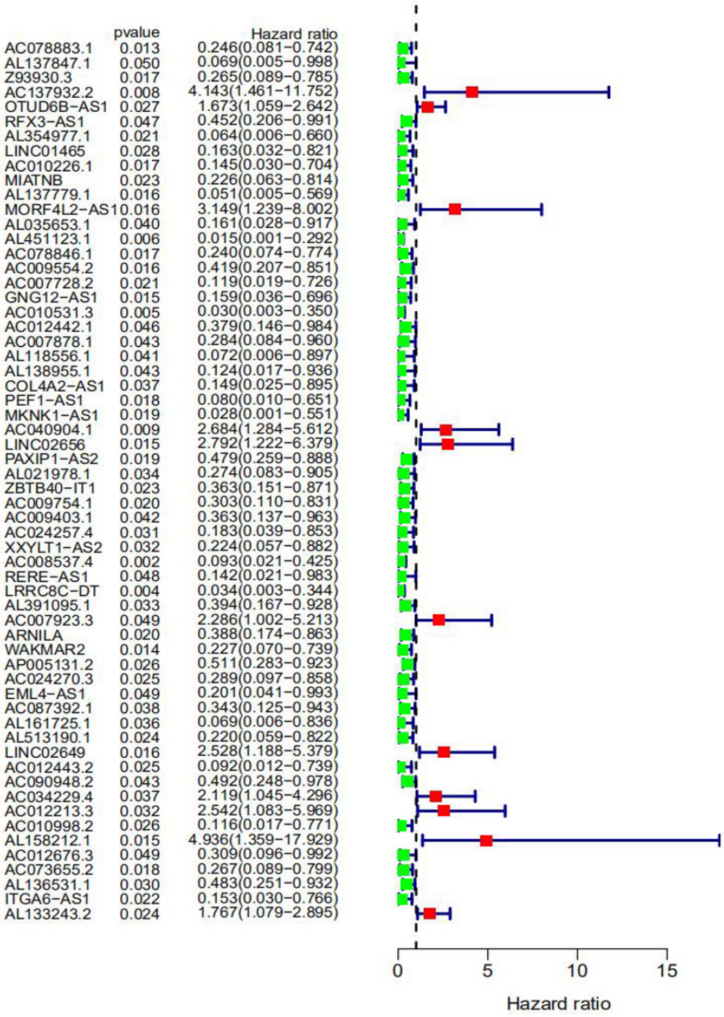

Supplementary Figure 2. LncRNAs were associated with high-risk group and low-risk group.

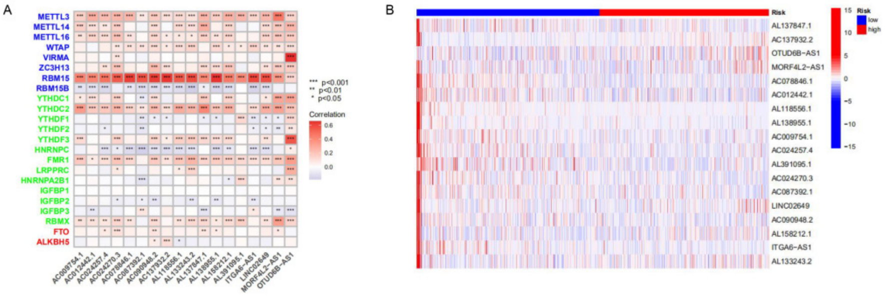

Supplementary Figure 3. Relationship between risk model related lncRNA and m^6 A-related-genes**(A)**. Differential expression of lncRNAs in different risk groups was analyzed by heat map**(B)**.

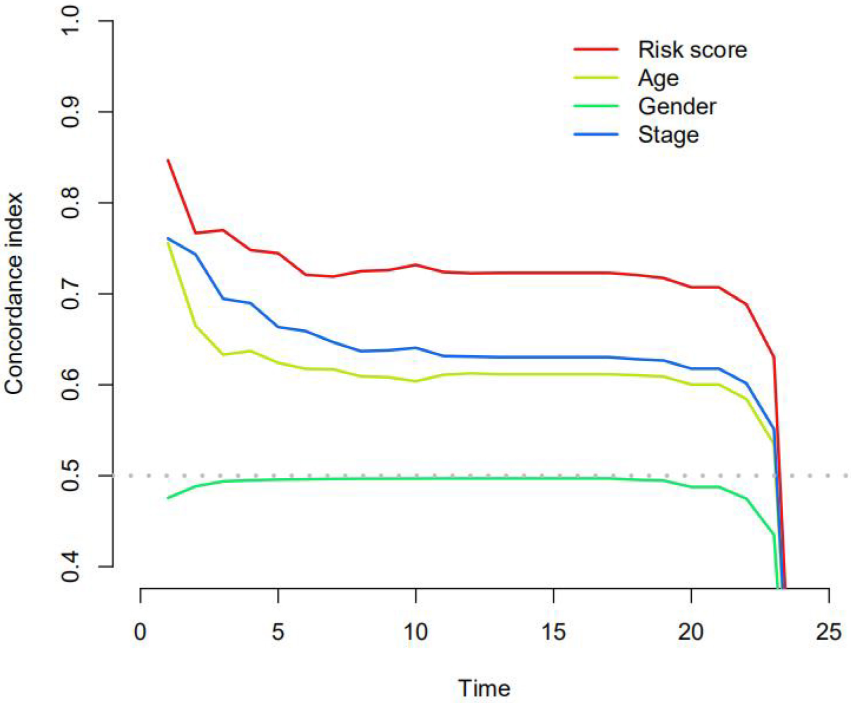

Supplementary Figure 4. C-index curve was used to compare various clinical factors (stage, gender, age, and risk score) as prognostic indicators.

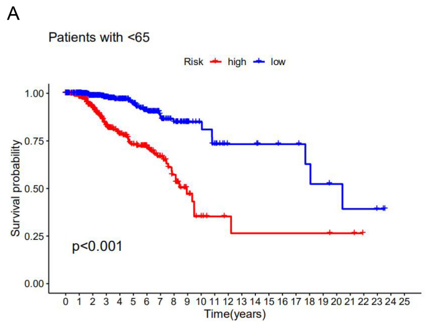

Supplementary Figure 5. Survival by age in patients older than 65 years and younger than or equal to 65 years in the high-risk group and low-risk group **(A, B)**.

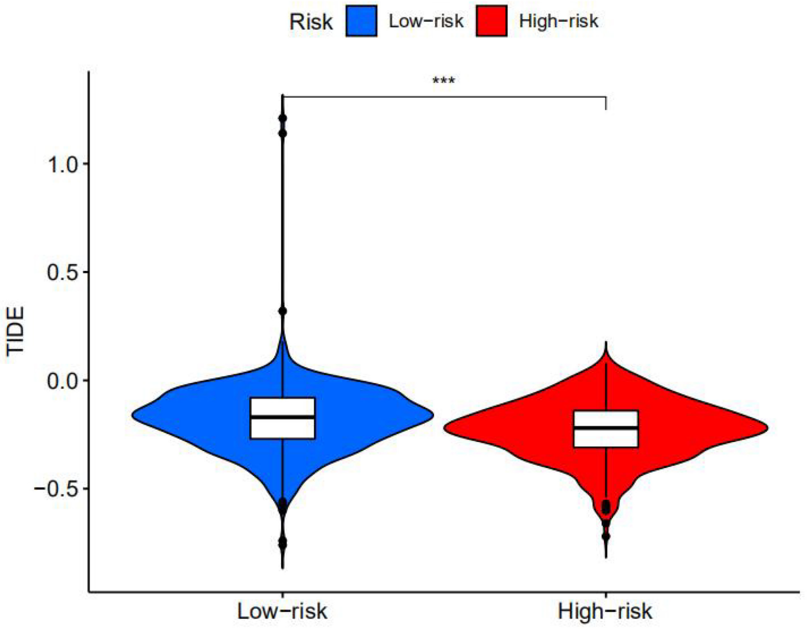

Supplementary Figure 6. TIDE database was used to determine the likelihood of immunotherapy and immune-escape in patients at high-risk group and low-risk group.

Supplementary Table 1. Clinical characteristics of BC patients in the TCGA database

Characteristic	BC patients (n = 1097)
**Vital status, n (%)**	
Alive	948(86.4%)
Dead	149(13.6%)
**Characteristic Age,n(%)**	**External validation cohort data(n=20)**
< 65	753(68.6%)
≥ 65	344(31.4%)
**AJCC-T stage,n(%)**	
T1	281(25.6%)
T2	635(57.9%)
T3	138(12.6%)
T4	40(3.6%)
Tx	3(0.3%)
**AJCC-N stage,n(%)**	
N0	516(47.1%)
N1	364(33.2%)
N2	120(10.9%)
N3	77(7.0%)
Nx	20(1.8%)
**AJCC-M stage,n(%)**	
M0	912(83.1%)
M1	22(2.0%)
Mx	163(14.9%)

Supplementary Table 2. Clinical characteristics of BC patients in the external validation cohort

**Characteristic Age,n(%)**	**External validation cohort data(n=20)**
< 65	17(85%)
≥ 65	3(15%)
**AJCC-T stage,n(%)**	
T1	12(60%)
T2	7(35%)
T3	1(5%)
T4	/
**AJCC-N stage,n(%)**	
N0	10(50%)
N1	6(30%)
N2	3(15%)
N3	1(5%)
**AJCC-M stage,n(%)**	
M0	20(100%)
M1	/

Supplementary Table 3. qPCR primer sequence

lncRNA	Species	Forward	Reverse
AL137847.1	Human	TATGCGAAATCCGCCAGACACC	TGAGCAACCCAGCATGTAGAGG
AC137932.2	/	/	/
OTUD6B-AS1	Human	AAGCTACGCGCTAGGCTCTT	GAGGTCCTCTGAAGCAGGGAA
MORF4L2-AS1	Human	GGCTAATGTTGACTGGAGTGT	AGAATGGAGATGAGGAAGGGTA
AC078846.1	Human	GTCTCATTGTCTGTGGTCTA	TCTCTTACTGTCTCCTGTCT
AC012442.1	Human	CAACACCATCACATTCAGAG	TAACATCAGCAGCAGAGTC
AL118556.1	Human	TCTCACTCTCCATCAACATC	CAACACTGCTCTTCCATTAG
AL138955.1	Human	AGGAGCCAGAAGACAGTAA	GCCAAGAGGAAGATAGTAGTAT
AC009754.1	Human	TCAAGGAGTTAGTGGTTCTG	TGGTTCTGGAGTGTAGCA
AC024257.4	Human	TGCCTCCTCTCCTCAATC	CCGCTAATACGACTCACTATA
AL391095.1	Human	CGTAAGAAGAGCAGGAGAG	GGACAGGACAGGATGGTA
AC024270.3	Human	TCGGAAGGCAGTGGAATA	AGGCAACAGAGTGAGAGT
AC087392.1	Human	GCAGTGGCTCTTGTAGTC	GGATGGTGTGGTTGATGG
LINC02649	Human	GCACGGTTGAGAACTATTG	GGAAGGCAGAGGTTGAAG
AC090948.2	Human	TAAGGTTGGAGGTAGTGAGA	CTGATGATGGTGATGAGAGTA
AL158212.1	Human	AATCCACAGCAGCATCAG	CCTCACTCTCAGCATCCT
ITGA6-AS1	Human	TTTCCATGAGGGAGGAGAGT	GCATCCCAGTATGATTCAGAAGTA
AL133243.2	/	/	/
Actin	Human	TGACGTGGACATCCGCAAAG	CTGGAAGGTGGACAGCGAGG

Supplementary Table 4. Prognostic model associated lncRNA coefficients and their relationship with high-risk and low-risk groups

lncRNA	Coefficient	Expression level association with prognosis
AL137847.1	-2.92970389342707	——
AC137932.2	5.13965984463825	high
OTUD6B-AS1	0.741301698570473	high
MORF4L2-AS1	3.51751820190694	high
AC078846.1	-2.00370072966735	low
AC012442.1	-0.953689283417208	low
AL118556.1	-2.6686256122187	——
AL138955.1	-2.68747823661317	——
AC009754.1	-1.27357342707535	low
AC024257.4	-1.66135470206632	low
AL391095.1	-0.972178666564357	low
AC024270.3	-1.73612202447013	low
AC087392.1	-1.16409248893925	low
LINC02649	2.00533615871912	high
AC090948.2	-0.986934051235532	low
AL158212.1	2.22204206572387	high
ITGA6-AS1	-2.11197312161232	——
AL133243.2	1.14956772567151	high
